# Healthy Life Centre participants’ perceptions of living with overweight or obesity and seeking help for a perceived “wrong” lifestyle - a qualitative interview study

**DOI:** 10.1186/s40608-018-0218-0

**Published:** 2018-12-06

**Authors:** Elin Salemonsen, Britt Sætre Hansen, Georg Førland, Anne Lise Holm

**Affiliations:** 1 Faculty of Health and Social Sciences, Department of Health and Caring Science, Western Norway University of Applied Sciences, Bjørnsons gate 45, 5528 Haugesund, Norway; 2 University of Stavanger, Faculty of Health Sciences, Kjell Arholmsgate 39, 4021 Stavanger, Norway

**Keywords:** Overweight, Obesity, Help-seeking, Personal responsibility, Healthy life Centres, Shame and pride, Qualitative research

## Abstract

**Background:**

Overweight and obesity are complex conditions, associated with a wide range of serious health issues. In contemporary society, body size is an important part of a person’s self-representation. Lifestyle changes are difficult and long-term weight management is associated with a high risk of failure. In primary health care in Norway, lifestyle interventions are offered by Healthy Life Centres (HLCs) to those seeking help with weight management. The aim of this study was to explore HLC participants’ experiences of living with overweight or obesity and perceptions of seeking help to change dietary and activity habits.

**Method:**

This exploratory study employed a qualitative design. Semi-structured in-depth interviews were conducted with 13 participants. Data were transcribed verbatim and analysed using qualitative content analysis.

**Results:**

The analysis resulted in one main theme: *Searching for dignity*, based on two themes: 1) *Needing to justify avoidance of personal responsibility* and 2*) A desire to change.*

**Conclusion:**

Changing dietary and activity habits is difficult as the emotional alternation between shame, guilt and pride influences the ability to assume personal responsibility. A deeper understanding of each participant’s perceptions and experiences is important for the ability to tailor and provide a high quality health service. Addressing participants’ emotional distress and search for dignity is necessary for enabling dietary and activity change. This should be considered in the future development of HLCs and health promotion interventions in order to educate service users about emotions and the role they play in food consumption and inactivity. Weight stigma at individual and system level as well as responsibility related to dilemmas about “right” or “wrong” lifestyle should be addressed.

## Background

Overweight and obesity present an increased risk to health and are major risk factors for a number of non-communicable diseases (NCDs) including type-2 diabetes (T2DM), cardiovascular disease (CVD), chronic obstructive pulmonary disease (COPD), some types of cancer, musculoskeletal disorders and mental health problems [1–3]. The risk of NCDs is primarily driven by tobacco use, physical inactivity, unhealthy diet and alcohol abuse [2]. The Norwegian Directorate of Health recommends the establishment of Healthy Life Centres (HLCs) in all municipalities in order to assist persons at risk of NCDs, or in need for support for health behaviour change or weight management [4]. Empirical evidence highlights the importance of lifestyle change as a key component of risk reduction and the promotion of healthy development [5]. A healthy lifestyle is associated with protective risk factor levels and lower levels of symptoms and illness, including psychological illness [6]. The outcome of educational interventions aimed at increasing the level of physical activity (PA) with follow up in primary care is uncertain [7]. In addition, a quantitative study of HCL lifestyle interventions reveals that participation in a prescribed group-based exercise programme for 3 months may improve physical fitness and Health Related Quality of Life (HRQoL) [8]. In a literature review, the main barriers to the success of educational programmes were found to be psychological and environmental, but also socio**-**economic [9].

The individualization of health has been discussed in recent decades and this ideological shift in health promotion makes every individual responsible for her/his own health and for learning to adopt rational lifestyle behaviours [10, 11]. Studies of attitudes towards obesity show that as many as 72% mentioned individual reasons for obesity. Media and public health campaigns may solidify beliefs that obesity is due to individual causes, thus increasing the stigma [12]. A Norwegian study of normative newspaper messages on obesity and health, revealed a focus on bodily conformity**,** linking leanness with attractiveness, obesity with ugliness and lack of control with lack of responsibility [13]. Numerous studies have documented harmful weight-based stereotypes such as persons with overweight and obesity are lazy, unintelligent, have poor willpower and are noncompliant with weight-loss treatment [14, 15]. These stereotypes give rise to stigma, prejudice and discrimination against obese persons in multiple domains of life [15, 16], thus contributing to their lower psychological functioning and well-being [17]. Stigma can be a barrier to seeking help for weight management [18, 19].

In a qualitative study of primary care physicians and nurses responsible for lifestyle change, a majority reported that a major barrier was patients’ unwillingness to change their habits [20]. In earlier studies of participants’ perceptions of self-responsibility in an intervention to prevent T2DM, the informants had an ambivalent attitude towards self-responsibility and their own role in lifestyle change and its maintenance [21]. A majority of lifestyle intervention participants characterize lifestyle change as a constant struggle [21, 22] and weight management as an endless battle against temptations [21]. In a study about lifestyle issues in clinical dialogues, patients took a proactive role in defending themselves against shame and made great efforts to present themselves as responsible agents in matters of health [23]. The participants in a previous qualitative study of Norwegian HLCs described how earlier life experiences and emotional baggage can influence lifestyle change and questioned whether or not HLCs can actually help participants with substantial emotional baggage to change their lifestyle [22]. In addition, HLC stakeholders have a wide range of expectations and they describe HLCs as a concept in development [24]. In a newly published cross-sectional study of the characteristics of participants who started attending a HLC, the most frequent reasons were being overweight, wanting to increase physical activity, improve dietary habits and cope with musculoskeletal health challenges [25].

The scientific evidence of the long term effects of health promotion interventions in primary care is still not convincing [9, 26, 27]. There are few studies of lifestyle interventions in HLCs in Norway and sparse knowledge of the participants’ background, and help-seeking needs. There is a need for a better understanding of the participants’ experiences and perceptions in order to understand how HLCs can provide a qualitatively good health service and support them to change their lifestyle. More research is needed to understand their perspective, as well as the more complex, less articulated influences such as knowledge, skills, motivation and emotional status that can lead to weight gain and inactivity, but also to change. The aim of this study was therefore to explore HLC participants’ experiences of living with obesity and perceptions of seeking help to change dietary and activity habits.

## Method

### Design

We chose a qualitative, descriptive and interpretative design grounded in hermeneutic methodology and tradition [28, 29]. The purpose of a qualitative approach is to explore complex phenomena and discover themes or patterns based on experiences and perceptions to understand behaviour in order to inform clinical practice [30].

### Study context

The Norwegian healthcare system provides health promotion in primary healthcare. HLCs constitute an interdisciplinary primary healthcare service providing effective, knowledge-based measures for people with, or at high risk of disease, who need support for health behaviour change and to cope with health problems and chronic disease [31]. This low threshold service is easily accessible through direct contact or by referrals from general practitioners (GPs) and participation is not based on Body Mass Index (BMI). At the HLCs, health education is provided by healthcare professionals (including physiotherapists, public health nurses, psychiatric nurses and bachelors in public health) to help participants change their lifestyle habits. The health conversation is based on each participant’s perception and understanding of the challenges for which she/he is seeking help. Physical activity in the form of individual or group-based in- and outdoor activities, often with a physical therapist, is offered two to three times a week. HLCs also have a healthy diet course consisting of 4 to 5 two-hour sessions with practical tasks and theory, often with a public health nurse or nutritionist. An intervention lasts for 3 months with the possibility to extend it on two occasions, although this is practiced differently in the various municipalities. The organisation of the HLC differs between the various municipalities and small communities have inter-municipal cooperation that enables participants to attend courses across municipal boundaries.

### Participants

Purposive sampling [32] was used to identify participants for interview to ensure that the sample included individuals of both sexes and various ages, from small and medium-sized municipalities, with experience of living with overweight or obesity. The inclusion criteria were; women and men aged 18 to 80 years with overweight or obesity, who had contacted the HLC to obtain help with weight management, and who were able to speak and understand the Norwegian language. HLC administrators were asked to send requests to service users who had participated in lifestyle courses. The participants in this study were recruited from five different HLCs in Norway. The first author contacted all the service users after they had consented to participate. A total of 13 participants were included in this study (Table 1). The majority had contacted the HLC on their own initiative because they wanted to change their lifestyle habits and lose weight. Some were recommended lifestyle changes such as regular moderately intensive physical activity, healthy diet and weight reduction by their GP. The participants were either overweight or obese and had additional challenges and diagnoses which put them at risk (Table 2).

**Table 1:** Participant characteristics

Characteristics	Number of participants
Gender	
Female	8
Male	5
Age	
30–69	13
Mean age women	47,5
Mean age men	55,4
Civil status	
Single/divorced	1
Wido*w*/widower	1
Partner/married	11
Education	
Secondary school	11
Bachelor degree or higher	2
Occupational status	
Employee 50–80%	4
Unemployed	2
Disability pension	5
Retired	2
Participation in HLC	
Healthy diet courses	11
Activity groups	12
Individual conversations with HP	13

**Table 2:** Self-reported challenges, strain and additional diagnoses (number of participants in brackets)

One or several of somatic diagnosis: type 2 diabetes (3), cardiovascular disease (CVD) (4), Chronic Obstructive Pulmonary Disease (COPD) (2), celiac disease (1), multi sclerosis (MS) (1), sleep apnoea (1), various chronic pain conditions (8), fibromyalgia (3), cancer (2)	
One or several of psychosocial strains and challenges: anxiety (3), depression (4), loss and grief (1), identity reactions (12), eating disorders (2), suicidal thoughts (2), alcohol abuse (1), isolation (6), financial difficulties (2)	

### Data collection

Individual semi-structured in-depth interviews were conducted to gather data on the service users’ perceptions of living with overweight or obesity and seeking help to change dietary and activity habits. ES and ALH developed the thematic interview guide with follow up questions in accordance with Kvale and Brinkman [33] and the first author (ES) performed the individual interviews over a five-month period in 2017. In accordance with the participants’ wishes, 11 interviews took place in the local HLCs and two at a university campus. After a presentation about the purpose of the study each interview was tape-recorded. The form of the interview was open and the interviewer invited the participants to speak freely about their experiences. The main questions asked were; *What is your perception of changing dietary and activity habits* and *why did you contact the HLC and ask for help?* Other questions explored personal goals, their need for help and their challenges. The interviews, which lasted between 66 and 131 min, were transcribed verbatim by the first author (ES). ES, ALH and BSH discussed the data in relation to the participants’ descriptions and if, and to what degree, the data material could answer the research question. Information power as discussed by Malterud et al. [32] guided the sample size.

### Analysis

The theoretical framework of qualitative content analysis in this study is grounded in a data-driven inductive approach, conventional qualitative content analysis, described by Hsieh and Shannon [34] and a text-driven search for patterns as described by Krippendorf [35]. The analysis process in our study was systematic and the codes, categories and themes are strongly linked to the raw data [34, 36]. Data were analysed by the analytical steps in Qualitative Content Analysis (QCA) described by Graneheim & Lundman [37] and in the light of the methodological discussion by Graneheim, Lindgren & Lundman [36]. According to Graneheim and Lundman, categories present the manifest content of the text. A theme is a tread of an underlying meaning on an interpretative level and an expression of the latent content of the text [37]. Both the phenomenological description of the manifest content (categories close to the text) and the hermeneutical interpretation of the latent content (themes distant from the text) [36] was used in our study. In line with the QCA method, all authors read the anonymized transcripts independently to obtain an overall impression, after which they met to discuss the material. The text was analysed by searching for content that described the participants’ perceptions of living with overweight or obesity and seeking help to change their dietary and activity habits. Based on the discussion all authors agreed on preliminary themes and categories. The first author (ES) then coded the interviews according to these themes. The text was divided into meaning units, abstracted and labelled with a code. The whole context was considered when labelling meaning units and codes. The various codes were compared based on differences and similarities and sorted into categories, sub-themes and themes. Categories were identified through an iterative process of identifying, grouping and regrouping. The development of the main theme and themes based on sub-themes and categories took the form of a hermeneutic spiral [36, 38] in a back and forth discussion process between all the authors over a period of several months.

## Results

The analysis resulted in one main theme: *Searching for dignity*, which was based on two themes: *Needing to justify avoidance of personal responsibility* and *A desire to change.*

### Searching for dignity

The main theme reflected the participants’ experience of living with overweight or obesity and their perceptions of seeking help to change their dietary and activity habits. For all the participants, living with overweight or obesity impaired their body image and self-esteem, causing a negative self-representation of living a perceived wrong lifestyle. The participants was seeking help with lifestyle change at the same time as they felt shame about their body and not managing on their own, and guilt of not adhering to a healthy diet or doing enough exercise. They had earlier experience of losing weight and relapses, and experienced a constant struggle between a healthy lifestyle and pleasure. They felt a need to explain their weight gain and barriers to change. On the other side, they desired change and motivation and felt pride about taking initiative and ask for help, exhibiting willpower and discipline. The participants tried to balance protection and disclosure of self with pride for taking the initiative and responsibility for change in order to feel normal, accepted and worthy. Searching for dignity seemed to be a red tread throughout the data, themes and sub-themes. The main theme is based on two themes with associated sub-themes and categories presented in Table 3. We will elaborate further on the findings below.

**Table 3:** Overview of the main theme, themes, sub-themes and categories

Main theme: *Searching for dignity*		
Theme	Sub-Theme	Category
Needing to justify avoidance of personal responsibility	Strain and challenges as barriers to change	Grief, loss and identity reactions
		Strain and life challenges
		Stuck in old habits and deferral
	A constant struggle and negotiation between healthy living and pleasure	Knowledge of healthy living
		Recognition of health risk and consequences
		Lack of willpower and relapses
		Expression of personal responsibility
	Feelings of shame, guilt or discouragement affect weight management	Negative feelings related to body image, self-esteem and confidence
		Feeling bad about not adhering to the diet or activity plan
		Lack of management in daily life
A desire to change	Health challenges and the need for improved self-respect trigger change	Personal goals to improve health, fitness, weight loss and better management of everyday life
		Health challenges, illness and risks
	Pride in self-management	Taking the initiative to change and asking for help
		Maintaining new habits and exhibiting willpower
	Hope, self-efficacy and meaningfulness increase motivation	Hope and self-belief
		Meaningfulness in life

### Needing to justify avoidance of personal responsibility

The participants’ need to justify avoidance of personal responsibility was based on the sub-themes *Strain and challenges as barriers to change* as well as *A constant struggle and negotiation between healthy living and pleasure*, and *Feelings of shame, guilt and discouragement affect weight management.*

#### Strain and challenges as barriers to change

It was important for the participants to explain their weight-gain and give reasons for why they ended up seeking help to change their lifestyle. Although the participants wanted to change their dietary and activity habits, they mentioned numerous barriers to change. These included grief, loss and identity issues*,* strain and life challenges such as depression, eating disorders, inactivity, alcohol abuse, suicidal thoughts, isolation, financial difficulties, being stuck in old habits and postponing things. Some of the participants were affected by their personal employment situation, changes in life, identity issues and the struggle to maintain their dignity and sense of worthiness. Only four were employed on a 50–80% basis, while the others were in receipt of a disability pension, retired or unemployed. One participant explained her loss of identity due to unemployment as follows:

*I identified myself with the job ... that was what made me. I worked in a kindergarten then and received a salary for what I did, but suddenly there was nothing. I felt like I was nothing. I could not go to work and could not manage what was normal for people to do ... self-image and everything…*(Female 50-59)

Another participant explained that he started to gain weight when he lost his job and developed serious depression:

*I was a CEO and then I was fired… and after that… I have had no work... because after that my world shattered.* (Male 60-69)

One participant described different kinds of grief reaction in response to the loss of his spouse and later his dog, stating that he isolated himself, started drinking, ate unhealthy food, just sat on the sofa and became inactive:

*I have to admit that after my wife died, I sat down… and then died ... we had a dog and the dog died too, and I couldn’t bear anything…and I didn’t go out…it was a voluntary isolation…*(Male 60-69)

The participants described being stuck in old habits, making multiple excuses, procrastination, deferrals and denial. Some of the participants expressed fear of losing control and stated that they put up walls to escape responsibility. One described how she intended to address the issue:

*I have really neglected the diabetes a little bit ... I have parked it on the stairs for a long time. It’s very hard to accept it! I have to learn to live with... that’s what it’s all about, right?* (Female 30-39)

#### A constant struggle and negotiation between healthy living and pleasure

This struggle can be described as negotiation between knowledge of healthy living and recognition of the health risks and consequences of being unable to resist the temptation of unhealthy food. It is a clear expression of personal responsibility to act upon this knowledge on the one hand and lack of willpower and discipline on the other. All the participants described their knowledge and understanding of healthy diets, the importance of being physically active and the relationship between lifestyle habits and illness. Their problem was not lack of knowledge and information but adhering to the new habits and changing routines. One of them expressed:

*We know what we should eat and what to do, but don’t act accordingly. That’s why we are gaining weight.* (Female 50-59)

Several participants found it difficult to be active and to resist unhealthy foods and some also described that lack of willpower to resist temptation is often reinforced by repeated efforts and relapses. The eternal struggle against temptation and pleasure was illustrated as follows:

*I have a problem with sugar. When I taste it, it’s hard …weaning is difficult. I think it’s the same mechanism as with alcoholics that you get hooked and you have to empty the bottle. That’s the way it is for me too, even if I’m stewed ... if there is more chocolate left in the bowl I cannot leave before its empty, even if I know I’ll get a headache and feel sick ... But anyway, sugar ... it’s not good, but for some reason I eat it... it is completely sick! (*Male 50-59*)*

*Sometimes you want to live your life too. To always be in focus and think about lifestyle change and think of exercise ... it’s quite tiring and a mental strain to be in focus in the long term.* (Female 30-39)

*I have to be aware of what I eat, right? It’s the first commandment to know what I’m eating, but I’m vacuuming the fridge in the evening, open the doors ... “is there anything good here” ... you know ... then you’ll figure out, right in the mine-field…* (Male 70-79)

The participants recognised the health risks, seriousness and future consequences of their situation. Two of the participants with T2DM explained:

*It’s almost five years since I got type 2 diabetes and if I don’t do anything about it… I know the consequences. Diabetes is actually quite serious and maybe I underestimate it a little because I ... the later harm and consequences are quite brutal. You can actually become blind, your legs can become numb, amputation ... these are the major aspects of having diabetes.* (Female 30-39)

*I knew if I didn’t turn over a new leaf I would never live to be an old man ... and I would like to ... at least live a little longer ... because the lifestyle I had, sitting still, diabetes, the imminent risk of a heart attack..*. (Male 60-69)

The participants blamed themselves for gaining too much weight and believed that they all have a personal responsibility to do something about their situation. Some of the participants suggested that people should take more responsibility for own health and that the health authorities should make more demands on the personal responsibility of people at risk and oblige them to participate in lifestyle interventions. One of them said:

*I thought when I was diagnosed with COPD... now I just have to start training... one can’t go around being so overweight with a COPD diagnosis ... it's hopeless ... but I did not start here only because of my COPD ... it's the weight ...it was clear…I can’t have it anymore ... I have to do something...* (Female 40-49)

#### Feelings of shame, guilt or discouragement affect weight management

Most of the participants had negative thoughts and ambivalent feelings about their body image, self-esteem and confidence, describing shame, discomfort and uncertainty related to their body. Many of them mentioned insufficiency in the areas of self, work, family, partner and children. It was evident that several of the participants had strong feelings about their body image and some of them expressed:

*I was afraid to go to the gym… pictures of slim and young people…I didn’t fit in with my big body…* (Male 60-69)

*At first it was incredibly difficult... it was scary ... I was pretty scared because there is prejudice against people with obesity … being at the gym, exercising, sweating and feeling uncomfortable, all of these ... and all these walls I’ve built up around myself ...* (Female 30-39)

Some experienced challenges related to sexuality:

*Overweight and sexuality are a combination that does not work well. Sexuality is a core of life that is quite important for many people, but there are many problems. I have a lot of complexes in relation to my sexuality ... and the desire for sex has become low because of the way I look and feel ... but that’s how I feel ... less nice, less appealing ... and it’s about putting yourself down ... self-image and self-esteem, because it’s part of yourself..*. (Female 30-39)

A clear sense of shame was described, both in relation to their own body, but also to not managing to lose weight and change to a healthier lifestyle by themselves. Some found asking for help difficult:

*Firstly, because you have to admit that you need help ... it becomes such a mental process to admit that you cannot quite manage it yourself ... and to train with others ... it was one of those barriers .... you pant and gasp ... I will never forget the first time ... knowing that others will see what bad shape you are in ... * (Female 40-49)

The participants had a great deal of experience trying to change their dietary and activity habits to lose weight. They described their negative weight loss experience and efforts as difficult due to constant relapses. Before starting at the HLC they had attended several “slimming programmes” and followed various diets, experiencing an initial weight loss but after a time gaining weight again. Several of the participants described feeling guilty for not sticking to their diet and activity plan. The challenge is to maintain the changes and make the new routines and habits permanent. After repeatedly slimming, dieting, gaining weight again and lack of a mastery experience, many participants experienced defeat, resignation and discouragement:

*I cannot say I did not wish there was an easier way to see progress. I saw it when I was training ... in the first two months I lost weight, but after that it stopped and I think stayed the same for three months… without losing anything… *(Male 30-39)

### A desire to change

The participants’ desire to change is based on the sub-themes *Health challenges and the need for improved self-respect trigger changes*, *Pride in self-management* and *Hope, self-efficacy and meaningfulness increase motivation.*

#### Health challenges and the need for improved self-respect trigger changes

All the participants had additional diagnoses (Table 2), which constitute a risk to their health. Their understanding of the severity and consequences triggered the turning point at which they made the decision to change their dietary and activity habits. They also had different personal goals and wishes for change. The desire to change to a healthier lifestyle was triggered by feedback from their body about health challenges, illness and risks, but also for preventive purposes. Several of the participants stated that they were limited in terms of the activities they could perform. Limitations in daily life, such as being unable to climb the stairs, play with grandchildren or participate in activities with one’s partner and children, make life more challenging and affect self-image. This desire to lose weight and be able to keep up with one’s children was expressed as:

*I have two youngsters at home…and I knew… when we were doing things together, I had to sit down because I was so tired. So my goal was to become more physically active… but it was that weight ... I knew I had to lose weight ... yes ...that was the starting point ...* (Female 40-49)

For several participants, better health, feeling strong, improved fitness and managing everyday tasks was most important. Others had additional needs and a desire for change, such as to control their blood glucose and reduce their medication. Some of the participants wanted to keep their job or be able to work again, while for others it was just about surviving. One participant stated:

*My goal is to survive ... quite simply. Get rid of those negative thoughts as well by being active ...* (Male 60-69).

One young participant described his need for normality and independence:

*That’s my reason…, basically, I may never get rid of it ... but the point is that I wish to get into such good shape that I no longer need the c-pap machine … that’s my goal…* (Male 30-39)

The desire to lose weight was described by all participants as a means of achieving a feeling of normality, acceptance and worth. One of them stated:

*There is a big body-focus in society. You actually feel less worthy when you are overweight and it’s very tiring. I’ve probably just felt it myself yes... but I feel it ... yes ...* (Female 40-49)

#### Pride in self-management

Recognition of the health risks, seriousness and consequences of their situation led them to take the initiative to ask for help and do something about their situation. Taking responsibility for one’s own health and changing dietary and activity habits leads to pride. Participants taking the initiative to change and asking for help, adhering to new habits and exhibiting willpower and discipline, expressed this pride directly and indirectly. Most of the participants presented themselves as responsible and explained how they took the initiative to change. They had read about HLCs and searched the internet for information and help, several of them had asked their GP for a referral to the HLC. Some of the participants emphasized their ability to maintain new habits and what they had experienced and managed to change in their diet and daily activity level. Even if some of them did not succeed in changing their dietary habits, they had a need to describe how they changed their activity habits or other things.

*My goal was to quit insulin injections before Christmas last year and I managed it in November… in the hospital they said that I was one of the few who had followed their advice.* (Male 60-69)

Several of the participants found it necessary to highlight what they actually managed and were proud of, how they took care to do their best. Willpower and discipline are two of the significant aspects that the participants described as necessary to successfully change habits. One participant described his willpower as follows:

*When I started cycling I had to turn back after 500 m because I was so out of breath. The following day, it was only sheer willpower that made me get on my bike. However, within a week, I went from cycling 500 meters and having to turn back, to cycling halfway to the city, which is 6 km! Now that's pretty good progress ...* *(laughter)* (Male 30-39)

One of the participants showed pride and willpower by describing how he performed despite the fact that he did not enjoy it:

*I've been spinning before and did not like it then, so I saw no reason that I'll like it now. However, I joined in and never missed a lesson. Nevertheless, I hate it as much now as I did before…but it works.* (Male 60-69)

#### Hope, self-efficacy and meaningfulness increase motivation

The participants believed they would manage to change their activity and dietary habits, while some even believed they could lose weight using their own resources. They were of the opinion that such change would increase their quality of life. All of them found motivation in self-management and mastering small changes, as well as management of daily life and everyday tasks. For several participants, voluntary work became a meaningful part of their life. Some of the participants had already experienced a change in strength and fitness, which they described as making them more socially active, leading to a feeling of hope, self-efficacy and well-being, which in turn strengthened their motivation to continue implementing lifestyle changes.

*It’s about those little things, the small milestones and steps and they are just as important as the chocolate I could enjoy after three months or ... Being able to go and work out with my daughter ... they are the important things ... to be able to do. I feel now ... now there's a lot more inner drive ...* (Female 40-49)

Several of the participants described their hope and belief that someday, in one way or another, they will manage to change their dietary and activity habits to achieve a healthier lifestyle. No one perceived this as simple or easy, but hope is important for remaining motivated.

## Discussion

This study explored how HLC participants experienced living with overweight or obesity and their perceptions of seeking help to change their dietary and activity habits. Below, we discuss the findings in the context of existing literature within this field.

The results suggest that the participants are basically seeking dignity to gain a better self-image and maintain their integrity. Several of the participants described low self-esteem and mental health issues, while some were painfully aware that they weigh too much and their weight issues reflect a deep sense of unworthiness. The desire to change may be seen as a wish to be normal and thereby feel worthy. Dignity can be related to self-esteem, as it refers to the worth of human beings, the right to be valued and respected. According to Schopenhauer, dignity is the opinion of others about our worth, while a subjective definition of dignity is our fear of others’ opinion [39]. This can illuminate the basic search for dignity in all human beings and in particular for persons with overweight and obesity.

There are a number of possible explanations for this search for dignity. It can be seen as a response to the stigma linked to being afflicted by overweigh or obesity. Goffman describes stigma as a deviation from our expectations of normality [40]. When reporting how they experienced living with overweight or obesity, the participants in our study described negative feelings related to body image, self-esteem and confidence (feelings of shame), feeling bad about not adhering to the dietary and activity plan (guilt) and lack of self-management in daily life (discouragement). This is consistent with previous studies showing that persons afflicted by overweight or obesity perceive that they are less worthy and experience a great deal of guilt, discouragement and shame [14, 23, 41, 42]. Several previous studies have described lifestyle change as an eternal struggle [22, 43], leading to feelings of unworthiness [14, 42, 44].

The participants in our study tried to explain the reasons behind their weight challenges and why they find change so difficult. In some cases it was a reaction to grief, the loss of a spouse, while others perceived challenges to their identity related to losing a job or being diagnosed with a chronic disease. These findings are in line with earlier studies and support the view that changing lifestyle habits is difficult as psychological and emotional distress can influence the ability to change [22, 45–47]. Participants with complex challenges and insufficient coping strategies, many of whom suffered from mental health problems, often struggled with follow up [8, 24]. Our participants also reported previous experiences of attending slimming programmes and losing weight, but subsequently gaining weight again after the intervention period. Some of them experienced these efforts as a hopeless enterprise and the relapses as shameful. This struggle is supported in earlier studies that the risk of weight regain includes a history of weight cycling and relapses [48]. Grant and Boersma [44] suggest that it is better to understand the nature of the problem rather than change the person. As the dominant counselling approach in weight management programmes is based on behavioural or cognitive behavioural paradigms, the benefits of a psychodynamic approach would be worth further exploration.

Several of the participants in our study reported lack of discipline and willpower as a challenge, and blamed themselves for not having more control. Jallinoja et al. [43] suggest that no matter how self-disciplined individuals are, if the dilemma between pleasure and health are not disentangled, lifestyle change will only be short term. In general, the participants in our study stated that their personal responsibility for a healthy lifestyle and changing their own situation was important to them. These findings are supported by an earlier study, which revealed that participants in an intervention to prevent type 2 diabetes had an ambivalent stance towards self-responsibility, yet constructed themselves as responsible and knowledgeable pro-health persons [21]. This can be supported by Goffman’s argument that people are likely to present themselves in a light that seems favourable [49].

Self-conscious emotions like shame, guilt, embarrassment and pride play a central role in motivating and regulating almost all of people’s thoughts, feelings and behaviour, and differ from basic emotions because they require self-awareness and self-representation [50]. This issue of self-representation was very clear in our study and is in line with the study by Guassora, Reventlow & Malterud [23] where patients presented themselves as responsible in dialogues about lifestyle and tried to defend themselves against shame [23].

The participants in our study expressed and believed that their overweight or obesity was self-inflicted and considered themselves guilty of not eating healthy food and adhering to their dietary and activity plan. This is in line with previous studies where positive attributes are accorded to people who are healthy, while those who become ill or have a less perfect body are blamed and considered self-indulgent, lazy, unmotivated, lacking self-discipline, less competent or even irresponsible and immoral [13, 14, 41, 42]. The personal attributes assigned to persons with overweight or obesity highlight the victim-blaming that occurs [12, 42]*.* Brownell et al. [51] hold that the two most important words in the national discourse about obesity are personal responsibility and that the concept of personal responsibility for health is deeply ingrained in our culture and political system. They state that good health has become more than a means to achieving personal goals such as greater attractiveness and increased longevity, but symbolizes self-control, hard work, ambition and success in life. Inherent in this symbolism is the concept that the individual controls behaviour, which in turn controls health. [51]. This paradox of control places many people in an untenable situation whereby they feel guilty about failing to perform the ideal behaviours [42] and are ashamed when they become ill, as is the case with the participants in our study (T2DM, CVD, COPD). According to the review by Puhl & Heuer, obese persons are blamed for their weight and a common perception is that weight stigmatization is justifiable and may motivate individuals to adopt healthier behaviours [14]. However, stigmatization of persons with overweight or obesity threatens psychological and physical health [14–16, 41] and generate health disparities [16]. Findings in a study by Täuber et al. [17] suggest that weight bias internalization in the form of moral condemnation contributes to the lower psychological functioning and well-being of people with overweight and obesity. The participants’ perceptions of responsibility in our study are clear, but we suggest that this emphasis on personal responsibility may lead to even greater shame when people experience lack of management or condemnation from society.

The theory of these self-conscious emotions is described by Tangney [50], Tangney & Fisher [50] and Tracy, Robins & Tangney [52]. In relation to shame and guilt, they argue that shame involves negative feelings about the stable, global self («I am a fat person»), whereas guilt involves negative feelings about a specific behaviour or action taken by the self (“I didn’t try hard enough to lose weight”). When the attentional focus is directed towards the public self, such as being publicly exposed as incompetent, it becomes an embarrassment. The public self is always present because it reflects the way we see ourselves through the real or imagined eyes of others [52, 53]. Goffman [40] noted that every social act is influenced by even the slightest chance of public shame or loss of face and people worry about losing social status in the eyes of others.

Some of the participants described isolating themselves from the outside world. This isolation was partly due to depression, but also because of negative feelings attached to self-esteem and body image. According to previous literature, the immediate response to shame is often to retreat or make oneself as small as possible [54, 55], which may explain why some of the participants in our study isolated themselves. There is a tremendous pressure in post-modern Western society to be thin and have a specific body shape, which for many symbolizes self-control, discipline, hard work, success and ability to manage indulgence [42, 54]. In the self-esteem theory, Maslow [56] described self-esteem as a basic human need or motivation that reflects a person’s overall subjective emotional evaluation of her/his own worth. He claimed that all people have a need or desire for a stable and high evaluation of themselves and self-respect in the form of self-confidence, skills and capability. Ignoring these needs produces feelings of inferiority and helplessness, which in turn give rise to basic discouragement.

In general, the participants described their desire for change as feedback from their body. Some seemed to be motivated to change their diet and level of activity by their appearance or health challenges, while for others the desire for change was for preventive reasons. Several participants reported that the seriousness of the health challenges serve as an ultimatum if they want to live longer and achieve better health. Several of our participants were either at risk of or had already developed NCDs as reported in Table 2. This finding is consistent with a previous study by Følling et al. [22], which reported that 91% of the participants in a HLC lifestyle intervention had multi-comorbidities, such as overweight, obesity, T2DB, muscle- and skeletal diseases and psychological issues. It is also in line with previous findings from Samdal et al. [25] describing the reasons for attending a HLC as the wish to increase physical activity and achieve a healthier diet in order to manage overweight, obesity and multiple health challenges. Our study adds to the literature about the challenges involved in health promotion interventions for overweight and obesity. In addition, we add to the literature on help-seeking needs, the underlying importance of self-representation, integrity, acceptance and dignity.

The participants in our study described pride in self-management. They were eager to talk about their initiative to change and the fact that they themselves asked for help. Theories of pride explain that when it comes to motivating social behaviour, pride may be the most important human emotion [52, 57]. Our most meaningful achievements, both on an everyday and life changing level, are accompanied by a feeling of pride. It is likely that pride evolved to provide information about an individual’s current level of social status and acceptance. Self-esteem may be an important part of this process and the development of pride may be closely linked to the development of self-esteem [52]. In a study by Guassora et al. [23], patients described their achievements as matters of honour, shifting from problematic issues to achievements of which they were proud. Our study supports these findings and highlights the meaning of pride in people’s presentations of self*.* In addition, our study contributes to descriptions of how participants alternate between shame, guilt and pride and how these self-conscious emotions influence the ability to assume or avoid responsibility.

The turning point can appear when the individuals in question understand the severity of the situation and realise that after several attempts and mistakes doing things by themselves, they need help and support from others, as the participants in our study recognized. Asking for help may involve swallowing hubristic pride as described by Tracy & Robins [52]. Studies have shown that individual motivation to lose weight and perceived self-efficacy are associated with better weight loss and beneficial effects on physical health and life satisfaction [58, 59]. Bandura’s theory of self-efficacy, the belief in one’s own ability to manage different tasks and reach specific goals, is important for behavioural change [60]. Previous studies have shown that participants who contacted a HLC themselves more often expressed a will for lifestyle change and less often dropped out than those referred by GPs [8, 24, 25]. Although the participants in the study by Samdal et al. [25] had autonomous motivation, they suggest that interventions have to address impaired self-efficacy. Our findings suggest that the participants experienced hope and self-efficacy related to using their own resources, but most of all as a result of the support they experienced at the HLC, which in turn strengthened their motivation to continue implementing lifestyle changes.

### Trustworthiness, strengths and limitations

A qualitative design may provide insight into complex phenomena. In line with the hermeneutic approach employed in this study, there is always a possibility of ambiguity and different interpretations of the meaning of the text. Addressing aspects of credibility, dependability, transferability and confirmability to increase trustworthiness is important [29]. We argue that the credibility, confirmability and dependability of the findings were strengthened by coherently and systematically analysing data using inductive coding [34, 35] and categorization in the interpretation process (hermeneutical circle) [28, 38]. The discussion of the findings on several occasions, as well as the variation in the disciplinary backgrounds of the author and co-authors, who are public health nurses (ES and BSH), a nurse specializing in health education (GF) and a psychiatric nurse (ALH), enriched the analysis and increased trustworthiness and confirmability. The analysis and data interpretation were influenced by the authors’ preunderstanding, therefore the findings are a constructionist coproduction of the participants’ perception of reality [30, 33, 61]. The first author (ES) conducted the data collection and the co-authors read all the transcribed interviews in order to minimize potential bias. The first author (ES) has several years of clinical experience as a public health nurse and also worked in an interdisciplinary team helping children, adolescents and their families to change their dietary and activity habits. Her background provided a preunderstanding and experienced based knowledge of the research phenomenon, in addition to valuable insights concerning lifestyle change and social stigma. The second author (BSH) and third author (GF) also have experience of health promotion and health education, which provides a valuable and useful overview of health promotion perspectives. The fourth author (ALH) contributed understanding of and valuable insights into the psychological and emotional findings. Another strength of this study is the semi-structured in-depth interviews, which allowed the participants to focus on their needs and perceptions. Each interview lasted from 66 to 131 min, providing opportunities to elaborate on the questions and follow up the responses. The variety in age, gender and socioeconomic status strengthens the utility and transferability of the findings, which are enhanced by the rich descriptions of the context, the informants from five different HLCs in Western Norway, the data collection and analysis, as well as the inclusion of quotations from a number of participants.  Information power [32] guided the data collection in order to ensure a variety of perceptions. This paper meets the requirements of the COREQ [62] checklist.

The strength of our study is the contribution to knowledge of HLC participants’ experiences of living with overweight or obesity and seeking help to change their dietary and activity habits. As a relatively new health service, there is a need for a better understanding of this phenomenon in order to provide a high quality health service. The findings of the Finnish study, in which nurses and GPs reported that the participants’ unwillingness to change lifestyle behaviour was a major barrier to lifestyle change [20], are in contrast to our findings, where the participants themselves stated that it is not unwillingness but rather shame, guilt and ambivalence towards assuming responsibility that hinder them. The unwillingness reported by the nurses and GPs may be a result of stigmatization and prejudice. However, the fact that it differs from the perceptions of the participants in the present study is also an interesting issue, which highlights the importance of the service user perspective.

Some methodological limitations should be taken into account when interpreting the results. Providers recruited participants from ongoing lifestyle interventions, thus it is possible that the participants were selected because it was known that they were satisfied with the HLC programme. The participants may have also been influenced by the ongoing process of change, and participation in both dietary and activity interventions over a period of time prior to the interviews.

## Conclusion

This study explored HLC participants’ experiences of living with overweight or obesity and perceptions of seeking help to change dietary and activity habits. Being stigmatized and feeling shame and guilt for not managing to live a healthy lifestyle and have a normal body, have consequences for people’s psychological and emotional health and affect their quality of life. This study contributes to the descriptions of emotional distress experienced by persons afflicted by overweight or obesity. In addition, the findings add to previous knowledge by illustrating the impact of shame, guilt and pride, as well as demonstrating the complexities involved in assuming responsibility for changing dietary and activity habits. Self-conscious emotions such as shame, guilt and pride play a central role in motivating and regulating almost all of people’s thoughts, feelings and behaviour. It is therefore necessary to address these emotions in lifestyle interventions and offer treatment that takes account of such feelings. It is easy to forget the person living with overweight or obesity when both the person her/himself and the provider are mainly focused on weight loss treatment. Future HLC and health promotion interventions need to educate service users about emotions such as shame, guilt and pride, and their role in food consumption and inactivity so that people with overweight or obesity are able to regulate their intake of food and physical activity. We suggest that regaining positive self-esteem and dignity will lead to the ability to assume personal responsibility for achieving a better quality of life. Weight stigma at individual and system level and as well as responsibility related to dilemmas about the “right” or “wrong” lifestyle should be addressed.

## Abbreviations

BMI: Body Mass Index
COPD: Chronic obstructive pulmonary diseases
CVD: Cardio vascular diseases
GP: General Practitioners
HLC: Healthy Life Centres
PA: Physical activity
T2DM: Type 2 diabetes
